# Long Noncoding RNA HOTTIP Serves as an Independent Predictive Biomarker for the Prognosis of Patients with Clear Cell Renal Cell Carcinoma

**DOI:** 10.1155/2020/4301634

**Published:** 2020-05-14

**Authors:** Zhanxin Liu, Zichun Wang, Xiaoxiong Wang, Meisong Lu, Guang Chen

**Affiliations:** ^1^Pharmacy Intravenous Admixture Service of The First Affiliated Hospital of Harbin Medical University, Harbin, China; ^2^Department of Reproductive, The First Affiliated Hospital of Harbin Medical University, Harbin, China; ^3^Department of Neurosurgery, The First Affiliated Hospital of Harbin Medical University, Harbin, China; ^4^Department of Urology, The Fourth Affiliated Hospital of Harbin Medical University, Harbin, China

## Abstract

Several studies have indicated that HOXA transcript at the distal tip (HOTTIP) play important roles in the tumorigenesis and development of various cancers. We aim to investigate the expression and prognostic value of HOTTIP in clear cell renal cell carcinoma (ccRCC). A systematic review of PubMed, Embase, Medline, and Web of Science databases was performed to select eligible literatures relevant to the correlation between HOTTIP expression and clinical outcome of different cancers. The association between the HOTTIP level and overall survival (OS), lymph node metastasis (LNM), or clinical stage was subsequently analyzed. Survival analyses were performed in a large cohort of more than 500 patients with ccRCC from The Cancer Genome Atlas (TCGA) using bioinformatic methods. Seventeen studies with a total of 1594 patients with thirteen kinds of carcinomas were included in this analysis. The result showed that high HOTTIP expression could predict worse outcome in cancer patients, with the pooled hazard ratio (HR) of 2.34 (95% confidence interval (CI) 1.96–2.79, *p* < 0.0001). The result also showed that elevated HOTTIP expression was correlated with more LNM (OR = 2.61, 95% CI 1.91-3.58, *p* < 0.0001) and advanced clinical stage (OR = 3.57, 95% CI 2.58-4.93, *p* < 0.0001). We further validated that ccRCC patients with higher HOTTIP expression tend to have unsatisfactory outcomes both in the entire TCGA dataset and different clinical stratums, like age, grade, and stage. The tumor of those patients was associated with a larger size, easier to metastasis, advanced clinical stage, and a higher pathological grade. These findings suggested that increased HOTTIP expression might act as a novel prognostic marker for ccRCC patients.

## 1. Introduction

In 2017, approximately 63,990 new cases of renal cell carcinoma (RCC) were diagnosed in the United States and approximately 14,400 patients died of this disease [1]. Clear cell renal cell carcinoma (ccRCC) is the most common pathological subtype of RCC, which accounts for about 75% of all cases [2]. This disease is characterized by resistance to radiotherapy and chemotherapy [3], and the cases which reported to respond immunotherapy are very few [4]. Therefore, interest on identifying novel prognostic biomarkers has been increased to predict its biological behavior.

Noncoding RNAs (ncRNA) are defined as not translated into proteins, including long noncoding RNA (lncRNA), microRNA, tRNAs, snRNAs, and snoRNAs, which may play a crucial biological role in cell metabolism and survival [5, 6]. lncRNAs are the majority of RNA, which are more than 200 nucleotides (nt) in length [7]. Many lncRNAs have been found to be abnormally regulated in expression and involved in the development of various human diseases including cancers, such as hepatocellular carcinoma, bladder cancer, gastric cancer, prostate cancer and breast cancer [8–12]. Strong evidence indicates that lncRNAs play important roles in a numerous number of biological processes such as embryonic development, cell growth, tumorigenesis, invasion, and metastasis [13, 14]. Moreover, lncRNAs can function as valid biomarkers for the diagnosis and potential predictors of patient outcomes in many kinds of carcinomas [15, 16].

HOXA transcript at the distal tip (HOTTIP), a lncRNA transcribed from the 5′ tip of the HOXA locus, can coordinately activate HOX genes and silence tumor suppressor genesis [17]. It is significantly upregulated in various types of human cancer, such as tongue squamous cell carcinoma, hepatocellular carcinoma, gastric cancer, colorectal cancer, and pancreatic cancer [18–22]. Recently, some studies have reported the relevance of HOTTIP in cancer prognosis. For example, Li et al. verified that elevated HOTTIP expression in osteosarcoma was associated with an advanced clinical stage and distant metastasis. HOTTIP knockdown suppressed osteosarcoma cell proliferation, migration, and invasion in vitro [23]. However, few studies have investigated the relationship between the significance of HOTTIP expression and ccRCC.

In the present study, we performed a meta-analysis to investigate the relationship between HOTTIP expression and the survival in patients of several different kinds of cancers. However, the expression and function of HOTTIP in ccRCC and their clinical significance remain unknown. We utilized 505 samples from The Cancer Genome Atlas (TCGA) to assess the ability of HOTTIP to predict survival in ccRCC. Multivariate Cox regression analysis showed that HOTTIP was an independent prognostic biomarker. Then, stratified analysis was carried out in different stratums, like age, grade, and stage. We hope our study will improve the accuracy of doctors' prognostic judgments.

## 2. Materials and Methods

### 2.1. Meta-Analysis Search Strategy

We searched potentially eligible literatures through PubMed, Embase, Medline, and Web of Science databases (up to September 7, 2019) to locate articles. We used “HOTTIP” or “HOXA transcript at the distal tips” and “cancer” or “tumor” or “carcinoma” as the keywords for search. Reference lists of relevant articles were also reviewed to identify potential eligible studies.

### 2.2. Inclusion and Exclusion Criteria

Eligible studies included in this meta-analysis had to fulfill the following criteria: (1) studied patients with any type of carcinoma; (2) examined the relationship between HOTTIP expression and clinical prognosis; (3) availability data in the studies was enough to calculate HR, OR, and corresponding 95% CI for overall survival; and (4) published as a full paper in English. Publications were excluded based on any of the following criteria: (1) non-English papers; (2) duplicate publications; (3) unqualified data; and (4) nonhuman research, letters, editorials, reviews articles, case reports, or laboratory articles. Two reviewers independently assessed all the retrieved literatures, and discrepancies were resolved by discussions and consensus.

### 2.3. Data Extraction and Quality Assessment

Two investigators independently extracted the essential data from the included studies. According to the inclusion and exclusion criteria, the following information and characteristics were extracted: (1) first author's name, (2) journal name, (3) year of publication, (4) origin country, (5) tumor type, (6) sample size, (7) HOTTIP testing method, (8) cut-off values, and (9) HR of HOTTIP expression as well as corresponding 95% CI, (10) follow-up time, and (11) covariate adjusted in the statistical analysis. When only Kaplan-Meier curves were provided, we extracted data from the graphical survival plots and estimated the HRs using the method described by Parmar et al. [24].

Quality assessment was performed independently by two investigators using the Newcastle-Ottawa Scale. A total of eight aspects were estimated in each study: adequate case definition, representativeness of cases, selection of controls, definition of controls, comparability of cases and controls on the basis of the design or analysis, ascertainment of exposure, same method of ascertainment for cases and controls, and nonresponse rate. The full score was 9 stars, and high quality was defined as a study with ≥7 stars [25].

### 2.4. Retrieval of Data on Gene Expression Profiles and Clinical Profiles from TCGA

Data on gene expression and clinical profiles of ccRCC were collected from The Cancer Genome Atlas (https://tcga-data.nci.nih.gov/tcga/). A total of 505 samples with ccRCC were used for further analysis, after removing the patients without clinical information. 329 male patients and 176 female patients constituted all 505 patients diagnosed with ccRCC. The median age (years) was 60 y (range: 26 y-90 y) and the median follow-up time (days) was 1175 d (range: 0 d-4537 d). There are 249 patients from stage I, 52 patients from stage II, 122 patients from stage III, and 82 patients from stage IV, respectively.

### 2.5. Enrichment Analyses

To assess the functional implications of HOTTIP, we computed the correlation between HOTTIP expression and protein-coding genes using the Spearman test. Only the genes with *p* value < 0.05 and Spearman correlation coefficient in top 1% were considered as coexpression partner. Based on coexpression protein-coding genes, DAVID was used for GO functional and KEGG pathway enrichment analysis [26]. The enriched GO terms or KEGG pathways with the criterion of *p* value < 0.05 were considered a potential function of HOTTIP as previously described [27].

### 2.6. Statistical Analysis

The extracted data were analyzed using STATA software V.13.0. All the HRs with the corresponding 95% CI were used to calculated the strength of association between HOTTIP and clinical prognosis. Combined HRs or ORs were conducted using Cochran's *Q* test and Higgins *I*-squared statistic to evaluate the heterogeneity in this present meta-analysis. *p* < 0.10 or *I*^2^ > 50% was defined as heterogeneity. A random effect model was applied when heterogeneity was statistical (*p* < 0.10), while a fixed effect model was used in the absence of heterogeneity (*p* > 0.10). Publication bias was assessed using the Egger's test.

To identify the prognostic value of HOTTIP, all patients from TCGA were dichotomized into high-risk and low-risk groups using the median expression level as the cut-off point. To estimate overall survival, we used the Kaplan–Meier method and the log-rank test to determine whether there was a significant difference in survival between the two risk groups. Furthermore, multivariate Cox regression analysis was used to assess the independent contribution of the gene, with the gene expression level, age, gender, grade, and stage as covariates. Then, differences in patients' survival between these covariates were evaluated by the Kaplan-Meier survival analyses in the same way as for the entire TCGA dataset. The threshold derived from the entire TCGA dataset were directly applied to different stratums. All analyses were performed with software R.

## 3. Results

### 3.1. Characteristics of Eligible Studies

**Figure 1:**
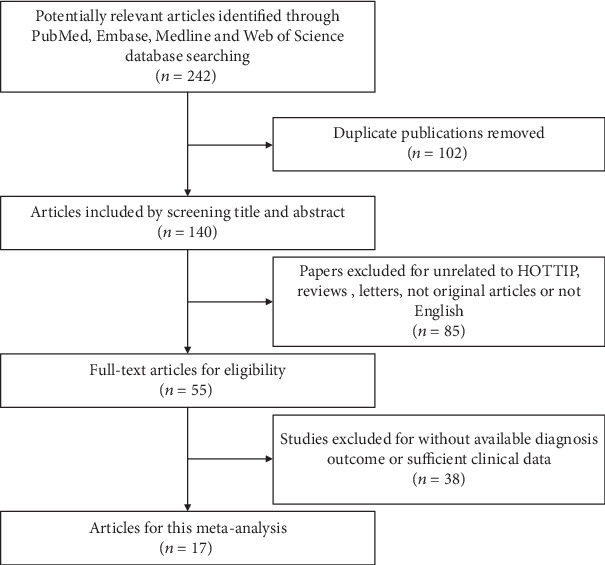
The flow diagram of this meta-analysis.

The main characteristics of eligible studies are summarized in Table 1. As shown in the flow diagram (Figure 1), there were 1594 cases with seventeen publications used to study HOTTIP expression between 2003 and 2019, fifteen studies for OS, eight for clinical stage, and eight for LNM [18–23, 28–38]. The types of cancers in the included studies were as follows: RCC, pancreatic cancer (PC), hepatocellular carcinoma (HCC), osteosarcoma, tongue squamous cell carcinoma (TSCC), colorectal cancers (CRC), gastric cancer (GC), breast cancer (BC), ovarian cancer (OC), prostate cancer, small-cell-lung cancer (SCLC), non-small-cell lung cancer (NSCLC), and periampullary region tumors (PRTs). One study used in situ hybridization (ISH) to measure HOTTIP expression; the others used qRT-PCR. No patient received chemotherapy or radiotherapy before surgery. According to the fold-change value, ROC curve, or median value, the patients were divided into two groups: high and low expression of HOTTIP.

### 3.2. Association between lncRNA HOTTIP and OS

**Figure 2:**
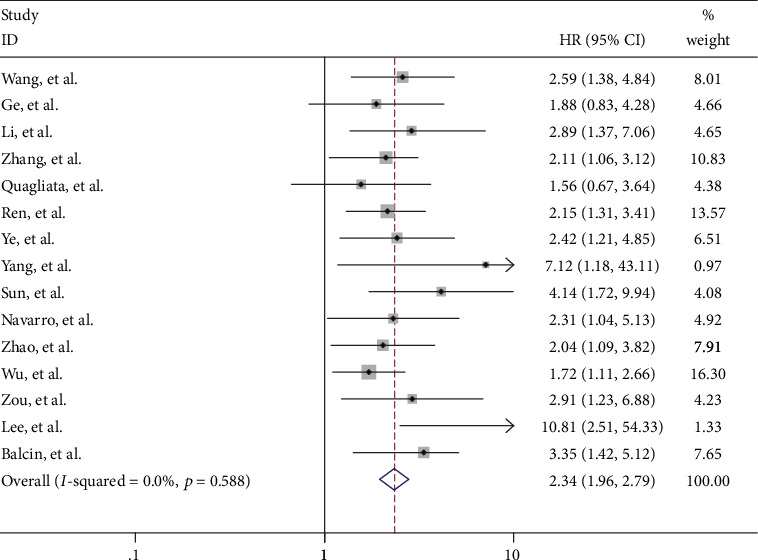
Forest plot of the pooled HRs of elevated HOTTIP expression for OS for the included studies.

Fifteen of the eligible studies including 1486 patients reported HR for OS according to different HOTTIP expression levels. The fixed effect model was used to calculate the pooled HRs with corresponding 95% CI as no significant heterogeneity was found among those studies (*I*^2^ = 0%, *p* = 0.588). According to meta-analysis result, we observed that the high HOTTIP expression group might be associated with a worse survival in various carcinomas (pooled HR = 2.34, 95% CI 1.96–2.79, *p* < 0.0001) (Figure 2).

### 3.3. Association between lncRNA HOTTIP and LNM

**Figure 3:**
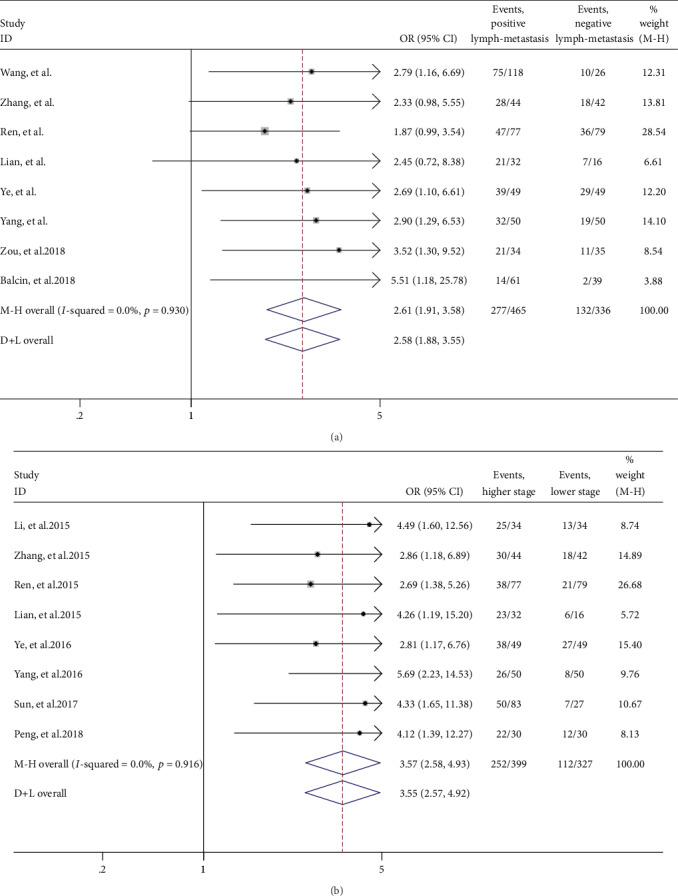
Forest plot for the association between HOTTIP expression levels with LNM notes (a) and clinical stage (b).

The association between the expression of HOTTIP and LNM was investigated in eight studies comprising 801 patients. The fixed effect model was applied as without heterogeneity (*I*^2^ = 0%, *p* = 0.93). The pooled OR was 2.61 (95% CI 1.91-3.58, *p* < 0.0001), which suggested that the elevated HOTTIP expression in tumor tissues was more prone to LNM (Figure 3(a)).

### 3.4. Association between lncRNA HOTTIP and Clinical Stage

For studies evaluating the association between HOTTIP expression and clinical stage, we used the fixed effect model to calculate the pooled HR with corresponding 95% CI because heterogeneity analysis showed that no between-study heterogeneity among those eight studies was found (*I*^2^ = 0%, *p* = 0.916). The pooled OR was 3.57 (95% CI 2.58-4.93, *p* < 0.0001), which revealed that patients with high HOTTIP expression in tumor tissues were more likely to lead to high clinical stage (Figure 3(b)).

### 3.5. Publication Bias

The publication bias of the present meta-analysis evaluated by the Egger's test indicated that there was evident asymmetry in this meta-analysis (*p* Egger′s = 0.003). And publication bias of the subgroup showed that small-study effects existed in the LNM group (*p* Egger′s = 0.018). However, sensitivity analysis by removing each research in turn showed the residual pooled HRs of OS was not impacted dramatically in Supplementary Table 1.

### 3.6. Expression of HOTTIP to Predict Overall Survival in ccRCC from Entire TCGA Dataset

**Figure 4:**
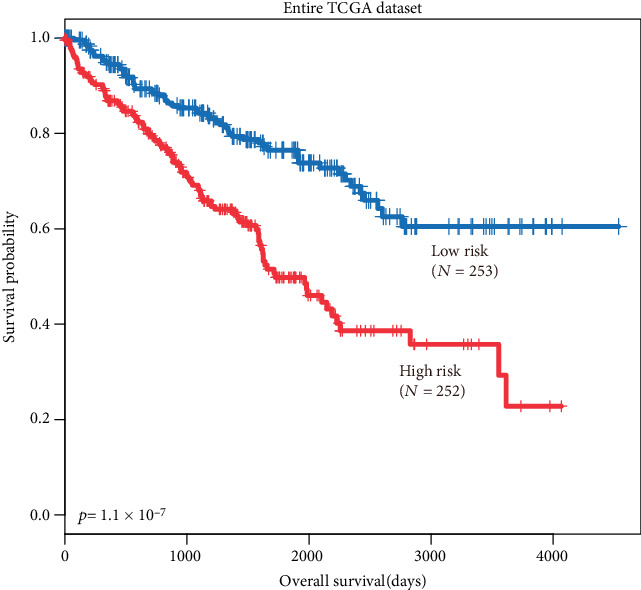
Prognostic value of HOTTIP in the entire TCGA dataset. The Kaplan-Meier plots show overall survival in the high-risk group (red) and the low-risk group (blue). The *p* value was calculated by the log-rank test. Overall survival was indicated in days.

As the meta-analysis showed overexpression of HOTTIP is associated with unfavorable survival in patients with various solid cancer, we wonder whether there are some connections between HOTTIP expression and ccRCC. Thus, we tested the prognostic value of HOTTIP in the TCGA dataset. 505 patients from TCGA were classified into low-risk (*n* = 253) and high-risk (*n* = 252) groups according to HOTTIP expression using the median expression value as cut-off. Survival differences between the low- and high-risk groups were evaluated with Kaplan-Meier survival curves. Our results showed that patients with a high risk had a significantly shorter overall survival (OS) time than those with a low risk (Figure 4). The overall survival rate in the high-risk group was 77% in 3 years, 62% in 5 years, and 58% in 10 years, whereas, the survival rate of the patients in the low-risk group was 87% in 3 years, 82% in 5 years, and 77% in 10 years, respectively.

### 3.7. Independence of Prognostic Value of HOTTIP from Other Clinical Variables

**Figure 5:**
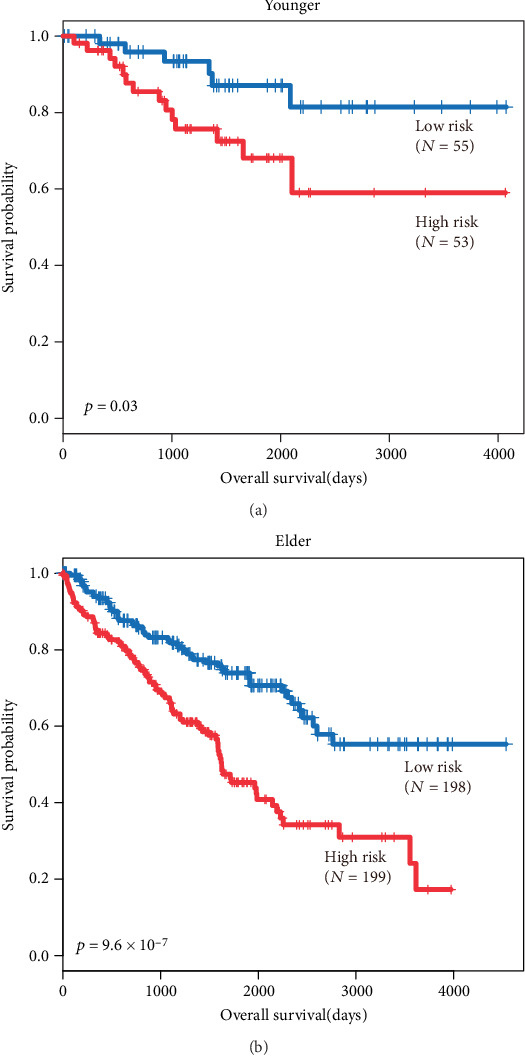
Subgroup analysis of the differentiation age. The Kaplan-Meier plots show overall survival in the high-risk group (red) and the low-risk group (blue). The *p* value was calculated by log-rank test. Overall survival was indicated in days. Notes: (a) Kaplan-Meier plots of younger patients (age < 50). (b) Kaplan-Meier plots of elder patients (age > 50).

**Figure 6:**
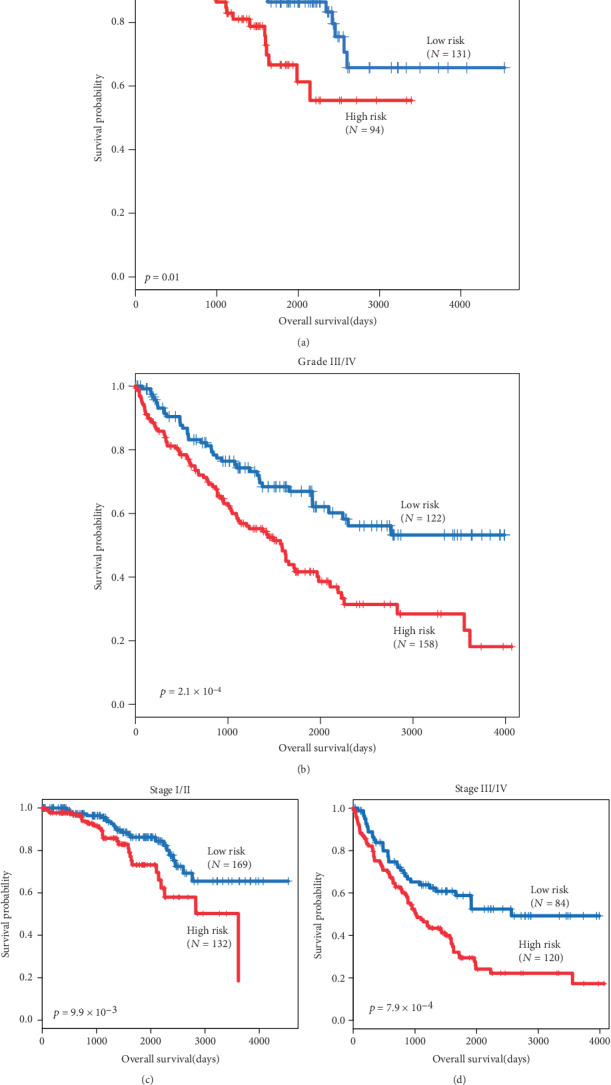
Subgroup analysis of the differentiation grade and stage. The Kaplan-Meier plots show overall survival in the high-risk group (red) and the low-risk group (blue). The *p* value was calculated by log-rank test. Overall survival was indicated in days. Notes: (a) Kaplan-Meier plots of grade I/II. (b) Kaplan-Meier plots of grade III/IV. (c) Kaplan-Meier plots of stage I/II. (d) Kaplan-Meier plots of stage III/IV.

For the goal of evaluating whether HOTTIP could distinguish the overall survival of patients in different groups, when age, gender, grade, and stage were taken into account, multivariate Cox proportional hazard analysis was performed. The results showed that age, grade, and stage were associated with OS, and the *p* value of HOTTIP expression was 0.01 (Table 2). To further investigate the ability of HOTTIP as an independent biomarker, stratified analysis was carried out. All patients were stratified into a younger stratum (age ≤ 50, *n* = 108) and an elder stratum (age > 50, *n* = 397). Both in elder and younger stratums, the results showed patients in the high-risk group had significantly shorter OS than those in the low-risk group (Figures 5(a) and 5(b)). The same methods were conducted in clinical factors, like grades and stages. Consist with the results before, the results showed that the lncRNA could further subdivide the patients into those likely to have longer survival and those likely to have shorter survival (Figures 6(a)–6(d)). Furthermore, we found that the tumor of patients with higher HOTTIP expression tends to be larger in size and to metastasize to distant organs, and the patients with a higher tumor grade and stage (Table 3). These results suggest that lncRNA HOTTIP can be the supplementary of current clinical prognostic system and improve the outcome by establishing a better therapy for patients with ccRCC.

### 3.8. Functional Analysis

**Figure 7:**
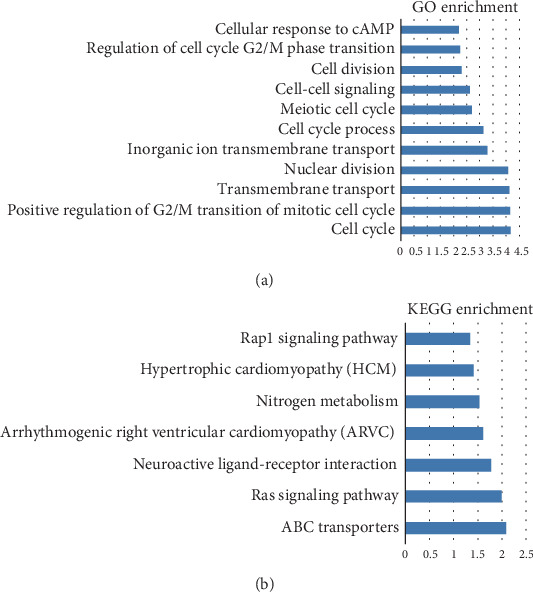
Enrichment analyses. The abscissa means the *p* values (log10 transformed) and the ordinate means typical cancer-related GO terms. Notes: (a) Gene ontology (GO) enrichment analysis of coexpressed protein-coding genes. (b) KEGG enrichment pathways significantly associated with the coexpressed protein-coding genes.

To gain the functional implication of HOTTIP in ccRCC tumorigenesis, we performed in silico analysis to infer potential functional roles of HOTTIP. The correlation was calculated between HOTTIP expression and protein-coding RNA expression in patients with ccRCC. The genes, with the criterion of *p* < 0.05 and the coefficients within top 1%, were regarded as coexpression partner of HOTTIP. A total of 940 protein-coding genes were significantly associated with HOTTIP. Then, GO function enrichment analysis was performed with these coexpression partners. It found that they clustered in the following items, including positive regulation of G2/M transition of mitotic cell cycle, the phospholipase C-activating G-protein-coupled receptor signaling pathway, chemical synaptic transmission, and cell division (Figure 7(a)). For the goal of digging out biology pathways, KEGG pathway enrichment analysis was performed as well. These genes are enriched in ABC transporters, the RAS signaling pathway, neuroactive ligand-receptor interaction, the Rap1 signaling pathway, etc. (Figure 7(b)).

## 4. Discussion

With the rapid development of genome-wide sequencing technology and completion of genomics consortiums, used to be considered transcriptional noise, lncRNAs have recently captured increasing attention [39]. Emerging evidence suggested that lncRNAs are involved in various biological processes, such as transcriptional regulation, epigenetic regulation, and posttranscriptional regulation [40–42]. Moreover, certain lncRNAs have shown tissue-specific expression patterns and exhibited unique profiles in a variety of cancers. These characteristics appear critical for their functional analysis and make it possible for application of lncRNAs as promising biomarkers in diagnosis and prognostic evaluation as well as treatment of cancer patients [43, 44].

The HOX gene is a class of genes that regulate embryonic development and cell fate. The HOX gene can be divided into four HOX clusters based on the similarity of the HOX sequences and the locus of chromosomes, as follows: HOXA (HSA 7, q23.3-q31.1), HOXB (HSA 17, q13.12-q13.13), HOXC (HSA 12, p15.2-p21.2), and HOXD (HSA 2, q21.31-q21.33) [45]. Monica et al. compared the expression of the whole HOX gene network in late-stage human fetal kidney development with the same patterns in normal adult kidneys and ccRCCs. They found that lumbosacral HOX genes systematically deregulated their expression in ccRCC and associated with kidney organogenesis. It seems that the HOX network acts a significant part in kidney carcinogenesis [46]. Wang et al. firstly identified that HOTTIP is located at the 5′ tip of the HOXA locus (chr 7p15.2), expressed in human fibroblasts such as those from the foreskin, foot, or hand, and is a 3764 nucleotide, spliced and polyadenylated lncRNA. So, it was named for “HOXA transcript at the distal tip” [17]. It has been confirmed that HOTTIP is associated with the WDR5/MLL complex to drive the histone H3 lysine 4 trimethylation (H3K4me3) and regulate the transcription of the distal HOXA gene locus during embryonic development [17]. Recent studies have demonstrated that HOTTIP was overexpressed in many cancers and played oncogenic roles in cancer progression. For example, Ge et al. identified that HOTTIP promoted HCC cell proliferation and metastasis, suggesting HOTTIP may serve as an oncogene in HCC and could be used as a prognosis predictor and a novel therapeutic target [18]. These studies suggested that HOTTIP might serve as an important prognostic factor in cancer patients.

In 2018, Peng et al. investigated the biological functions and molecular mechanisms of HOTTIP in RCC [34]. They found that HOTTIP could regulate cell growth and apoptosis by epigenetically silencing LATS2 in RCC. And HOTTIP expression was upregulated in RCC tissues compared with normal tissues. Moreover, a similar result was found between several RCC cell lines and the normal kidney cell line. They also observed that overexpression of HOTTIP was significantly associated with an advanced clinical stage (*p* = 0.018) and a larger tumor size (*p* = 0.004). However, the correlation between the expression level of HOTTIP and overall survival in RCC patients is still unknown.

We wonder whether there are more connections between HOTTIP expression and ccRCC. We performed survival analyses based on TCGA datasets. First, consistent with the results in the meta-analysis, patients with a higher expression level were associated with poor survival with the entire TCGA dataset. To further investigate the ability of HOTTIP as an independent biomarker, multivariate Cox regression analysis was performed. The results showed the expression of HOTTIP was an independent predictor, when age, gender, grade, and stage were taken into account. Then, stratified analysis was carried out in different stratums, like age, grade, and stage. All results suggested that patients with a high expression value of HOTTIP tended to have poor survival. Then, the GO and KEGG pathway enrichment analysis showed that significantly enriched pathways are targeted by HOTTIP in ccRCC involved in cell cycle, ABC transporters, and the RAS signaling pathway. It might reveal the functional role of HOTTIP as an oncogene in ccRCC and provide further studies with new thoughts. However, our study still has several limitations. For example, there is no experimental data to clarify the underlying molecular mechanisms of HOTTIP. Thus, in vitro and in vivo experimental studies of HOTTIP are needed to settle and enhance our understanding of ccRCC.

In conclusion, our results confirmed that expression of HOTTIP in ccRCC was correlated with overall survival and might be an independently prognostic marker for ccRCC patients. Hence, the elucidation of the underlying mechanisms about HOTTIP might help to advance our understanding of the biology of ccRCC, and further studies in this area are necessary to elucidate and confirm this association.

## Figures and Tables

**Table 1:** Main characteristics of the eligible studies.

Author	Year	Country	Ethnicity	Cancer type	Methods	Cut-off	Sample size	Survival analysis	Follow-up (total)	Covariants	NOS
Wang et al.	2015	China	Asian	PC	qRT-PCR	Fold-change	144	Multivariable analysis	60 Mons	LNM	7
Ge et al.	2015	China	Asian	HCC	qRT-PCR	NG	48	Kaplan–Meier curve	72 Mons	NG	7
Li et al.	2015	China	Asian	Osteosarcoma	qRT-PCR	Median	68	Multivariable analysis	60 Mons	Clinical stage, tumor size	7
Zhang et al.	2015	China	Asian	TSCC	qRT-PCR	Median	86	Multivariable analysis	60 Mons	LNM, clinical stage	7
Quagliata et al.	2014	Switzerland	Caucasian	HCC	qRT-PCR	ROC curve	52	Kaplan–Meier curve	80 Mons	NG	7
Ren et al.	2015	China	Asian	CRC	qRT-PCR	Median	156	Multivariable analysis	65 Mons	LNM, clinical stage	7
Lian et al.	2015	China	Asian	CRC	qRT-PCR	Fold-change	48	NG	NG	LNM, clinical stage, tumor size	7
Ye et al.	2016	China	Asian	GC	qRT-PCR	Median	98	Multivariable analysis	60 Mons	LNM, clinical stage, tumor size	7
Yang et al.	2016	China	Asian	BC	qRT-PCR	Median	100	Multivariable analysis	>100 Mons	LNM, clinical stage, tumor size	7
Sun et al.	2017	China	Asian	SCLC	qRT-PCR	NG	115	Kaplan–Meier curve	80 Mons	Clinical stage	7
Navarro et al.	2019	Spain	Caucasian	NSCLC	qRT-PCR	Mean + SD	99	Multivariable analysis	98 Mons	NG	7
Zhao et al.	2018	China	Asian	GC	qRT-PCR	NG	126	Multivariable analysis	60 Mons	NG	7
Wu et al.	2018	China	Asian	HCC	qRT-PCR	ROC curve	155	Multivariable analysis	>100 Mons	Tumor recurrence	7
Zou et al.	2018	China	Asian	OC	qRT-PCR	Median	69	Kaplan–Meier curve	60 Mons	LNM	7
Lee et al.	2019	Korea	Asian	Prostate cancer	ISH	NG	70	Kaplan–Meier curve	>100 Mons	Pathologic stage	7
Balcin et al.	2018	Turkey	Caucasian	PRTs	qRT-PCR	Fold-change	100	Multivariable analysis	77 Mons	LNM	7
Peng et al.	2018	China	Asian	RCC	qRT-PCR	Median	60	NG	NG	Clinical stage, tumor size	7

PC: pancreatic cancer; HCC: hepatocellular carcinoma; TSCC: tongue squamous cell carcinoma; CRC: colorectal cancer; GC: gastric cancer; BC: breast cancer; SCLC: small cell lung cancer; NSCLC: non-small-cell lung cancer; PRTs: periampullary region tumors; OC: ovarian cancer; RCC: renal cell carcinoma; qRT-PCR: quantitative real-time polymerase chain reaction; ISH: in situ hybridization; LNM: lymph node metastasis; NG: not given.

**Table 2:** Correlation between HOTTIP expression and clinicopathological features.

Variables	HOTTIP expression		*p* value
	Low *N* = 253	High *N* = 252	
Age			0.9137
≦50	55	53	
>50	198	198	
Tumor size			0.0003
>7 cm	104	144	
≦7 cm	149	108	
Lymph node status			0.103
Negative	118	108	
Positive	4	10	
Metastasis			0.0248
Negative	212	186	
Positive	30	47	
Grade			0.0012
I and II	131	94	
III and IV	122	158	
Stage			0.0011
I and II	169	132	
III and IV	84	120	

*p* value was determined using Fisher's exact test.

**Table 3:** Univariate and multivariate survival analyses for ccRCC patients.

Variable	Univariate analysis			Multivariate analysis		
	HR (95% CI)	Regression coefficient	*p* value	HR (95% CI)	Regression coefficient	*p* value
Stage	1.946 (1.695-2.233)	0.665	1.0 × 10 − 16			
Age	1.027 (1.013-1.040)	0.026	6.0 × 10 − 5			
Gender	0.990 (0.719-1.364)	-0.009	9.5 × 10 − 1			
Grade	2.394 (1.937-2.960)	0.873	6.6 × 10 − 16			
HOTTIP	1.679 (1.220-2.310)	0.518	1.4 × 10 − 3	1.521 (1.072-2.159)	0.419	1.0 × 10 − 2
Tumor size	3.008 (2.129-4.251)	1.101	4.1 × 10 − 10	2.063 (1.426-2.985)	0.724	1.0 × 10 − 4
Lymph node status	3.076 (1.587-5.962)	1.123	8.7 × 10 − 4	2.311 (1.149-4.645)	0.837	1.8 × 10 − 2
Metastasis	4.362 (3.172-6.000)	1.473	1.0 × 10 − 16	3.186 (2.235-4.541)	1.158	1.4 × 10 − 10

## Data Availability

All the data of the paper were based on a free database TCGA.

## Supplementary Materials

Supplementary Materials Supplementary Table 1: result of sensitivity analysis in the OS group.
