# Supplementary material for: Long Noncoding RNA HOTTIP Serves as an Independent Predictive Biomarker for the Prognosis of Patients with Clear Cell Renal Cell Carcinoma
Source: Int J Genomics. 2020 May 14;2020:4301634. doi: 10.1155/2020/4301634 (PMC7255047; doi:10.1155/2020/4301634)
Supplement: Supplementary Materials — Supplementary Table 1: result of sensitivity analysis in the OS group. [file 4301634.f1.pdf]

Supplementary Table 1: Result of sensitivity analysis in OS group

Study omitted	Estimate	[95% Confidence Interval]
Wang, et al. (2015)|	2.3150864	1.9248102 - 2.7844951
Ge, et al. (2015)|	2.3608572	1.9693037 - 2.8302629
Li, et al. (2015)|	2.3119111	1.9284929 - 2.7715597
Zhang, et al. (2015)|	2.3645585	1.9602613 - 2.8522403
Quagliata, et al. (2014)|	2.3795094	1.9853954 - 2.8518577
Ren, et al. (2015)|	2.3663821	1.955989 - 2.8628812
Ye, et al. (2016)|	2.3301847	1.9402601 - 2.7984705
Yang, et al. (2016)|	2.3106437	1.9340111 - 2.7606223
Sun, et al. (2017)|	2.2798145	1.9027573 - 2.7315907
Navarro, et al. (2019)|	2.3372765	1.9491453 - 2.8026958
Zhao, et al. (2018)|	2.363549	1.9653141 - 2.8424788
Wu, et al. (2018)|	2.4807923	2.0442615 - 3.0105395
Zou, et al. (2018)|	2.3133554	1.9304669 - 2.7721863
Lee, et al. (2019)|	2.2883053	1.9146935 - 2.7348197
Balcin, et al. (2018)|	2.2674465	1.885883 - 2.7262106
Combined |	2.3359258	1.9568589 - 2.7884225
